# Strategies and Best Practices That Enhance the Physical Activity Levels of Undergraduate University Students: A Systematic Review

**DOI:** 10.3390/ijerph21020173

**Published:** 2024-02-01

**Authors:** Chanté Johannes, Nicolette V. Roman, Sunday O. Onagbiye, Simone Titus, Lloyd L. Leach

**Affiliations:** 1 Department of Sports, Recreation, and Exercise Science, University of the Western Cape, Cape Town 7535, South Africa; sonagbiye@frederick.edu (S.O.O.); titusdawsons@sun.ac.za (S.T.); lleach@uwc.ac.za (L.L.L.); 2 Centre for Interdisciplinary Studies of Children, Families, and Society, University of the Western Cape, Cape Town 7535, South Africa; nroman@uwc.ac.za; 3 Department of Health and Exercise Sciences, Frederick Community College, Frederick, MD 21701, USA; 4 Centre for Health Professions Education, Faculty of Medicine and Health Sciences, Stellenbosch University, Cape Town 7505, South Africa

**Keywords:** physical activity, strategies, best practices, undergraduate university students, systematic review, participation, sustainable development goals

## Abstract

Significant numbers of undergraduate university students are not meeting the physical activity guidelines recommended by the World Health Organisation. These guidelines suggest that university students should aim for 150–300 min of moderate or 75–150 min of vigorous physical activity. Strategic interventions need to be implemented to address this global public health concern. The aim of this study was to review the strategies and best practices to enhance the physical activity levels of undergraduate university students. Utilising the PRISMA guidelines, electronic databases—PubMed, Science Direct, Academic Search Complete, ERIC, Web of Science, CINAHL, SAGE, and SPORTDiscus—were searched between September 2022 and February 2023 using terms and synonyms related to physical activity, strategies, best practices, and undergraduate university students. Studies were critically assessed for their quality using an adapted version of the CASP and RE-AIM frameworks. Eleven articles met the inclusion criteria for the review. The studies reported the use of social media platforms, mobile phone applications, web-based technology, online text messages, in-person classes, and an “exergame” as methods to increase engagement in physical activity. Findings from this review indicated that validated questionnaires emerged as the predominant measurement tool. Furthermore, the frequent use of social network sites served as a best practice for implementing and promoting physical activity interventions. It is recommended that universities promote health-enhancing physical activities based on current trends and strategies, such as technology-based interventions and the use of social media, that are relevant to contemporary university students.

## 1. Introduction

Physical inactivity has been identified as the fourth leading risk factor for global mortality and was estimated to cause 6% of deaths worldwide [1]. Currently, one in four adults are not meeting the global physical activity (PA) recommendations [2]. Consequently, individuals who are not physically active do not reap the health benefits associated with regular engagement. Research shows that there are benefits for students that can be derived from engagement in regular PA [3,4]. These benefits include lowered levels of cardiovascular disease and diabetes and the reduced possibility of weight gain, stress, depression, anxiety, and sedentary behaviour [4,5,6]. It is believed that, when compared to sedentary people, active individuals have better control over high-risk comorbidities [7]. With the high prevalence of physical inactivity among young adults—such as undergraduate university students—interventions, strategies, and guidelines must be implemented by relevant professionals in higher education institutions to address this health concern [8].

Physical inactivity has been examined as a prevalent trend among young adults of which undergraduate university students are classified [9,10]. Studies worldwide have reported high rates of insufficient PA among university students [11,12,13,14], for instance, 65% of Canadian university students are not meeting the PA guidelines for health, and approximately 41.4% of undergraduate students in Malaysia are physically inactive, with only 0.3% participation [12,15]. Furthermore, one in three university students in Romania have been found to be inactive [16]. These studies demonstrate low rates of PA which highlight the need for the development of interventions to address physical inactivity among university students. However, before student-specific PA interventions can be developed, research is needed to explore the strategies and practices that may be suitable to enhance the PA levels of undergraduate university students.

A previous systematic review that focused on interventions to increase PA indicated that to enhance PA levels, emphasis should be placed on interventions that target behavioural components, such as self-monitoring, goal-setting, and rewards and incentives [17]. Studies have focused on determinants of behaviour such as psychosocial factors that may influence PA participation [9,17,18]. This evidence suggests that techniques to change behaviour may be a useful strategy to enhance short-term PA engagement but that this engagement is seldom maintained [18].

Previous researchers have examined the effectiveness of health promotion strategies and interventions on university students, mainly the effectiveness of practices focused on stress [19], weight control, and food intake [20], in addition to the development of technology, video games, and social media [21,22]. However, limited evidence exists on how these PA strategies are implemented in the university environment, how these strategies are utilised and how these tools are used to measure participation in PA [23]. A previous study that investigated a video game-based PA intervention focused solely on randomised controlled trial studies, therefore limiting additional research through different methodologies such as qualitative and quantitative methods [21]. Additional literature reporting on the strategies used to promote PA is required, specifically focusing on the methodology of the interventions [24].

With the above-mentioned limitations having been observed in previous reviews, the present systematic review aimed to synthesise the existing literature on the strategies and best practices that enhance the PA levels of undergraduate university students. Strategies to improve participation in PA involve approaches, such as data collection methods, used to generate a plan to enhance engagement among students. Best practice focuses on the type of intervention found to be the most effective in enhancing the PA levels of undergraduate university students. As a result, these terms were used to generate a holistic view of the previous literature considered to be effective in increasing engagement in PA in a university environment. These results may provide insight into the strategies and best practices that may be used to enhance the PA levels of undergraduate university students.

## 2. Materials and Methods

**Figure 1:**
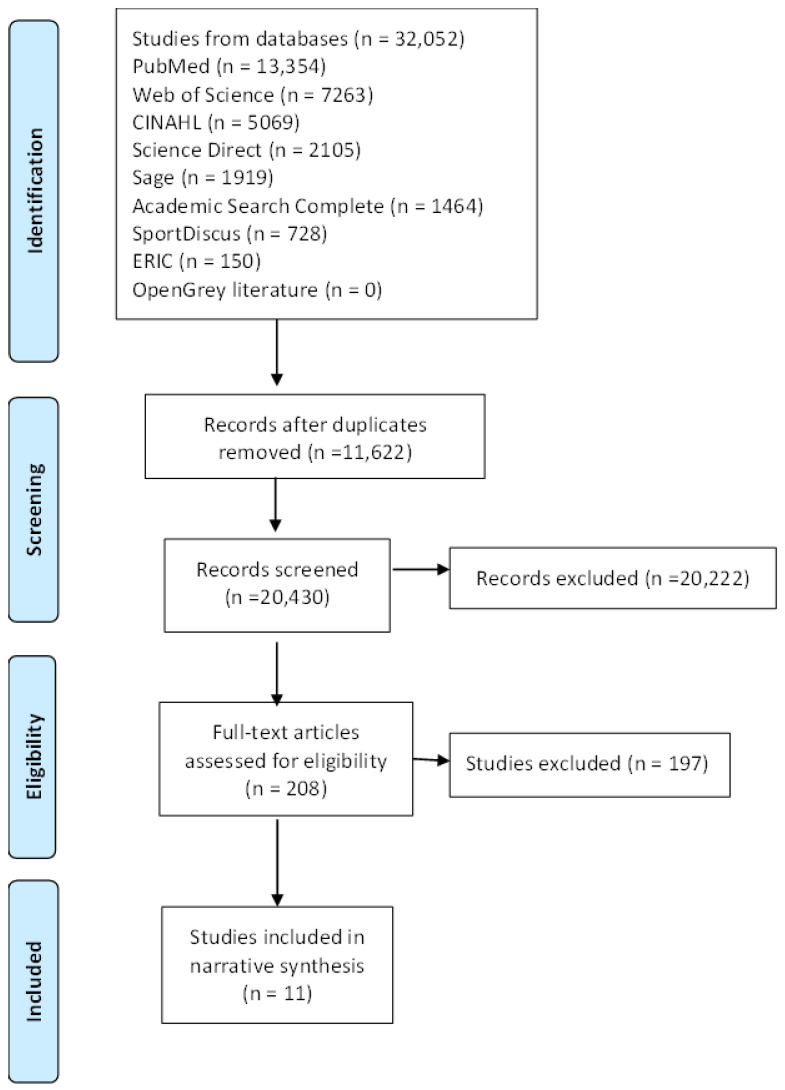
PRISMA flow diagram of included studies.

Each phase of the screening processes was completed in accordance with the Preferred Reporting Items for Systematic Reviews and Meta-Analyses (PRISMA) standards for systematic reviews, which are illustrated in a flow diagram [25] (Figure 1). Details of the full protocol registration for this systematic review may be found on the Open Science Framework (protocol reference: 10.17605/OSF.IO/BV73G).

### 2.1. Search Strategy

Electronic databases were searched between September 2022 and February 2023. This included PubMed, Science Direct, Academic Search Complete, ERIC, Web of Science, CINAHL, SAGE, and SPORTDiscus. The grey literature was searched by entering terms into OpenGrey. The search terms were as follows: ((physical activity) AND (exercise)) OR (strategies) OR (best practices) OR (interventions) OR (programmes) OR (programs) OR (approaches) AND (young adults) OR (university students) OR (college students) OR (undergraduate students). Full information on database-specific search strategies can be found in the Supplementary Materials (Supplementary File S1). The same keyword variations were used for all nine databases. In addition, the reference lists of the retrieved articles were manually searched for potentially eligible studies.

Outputs from the various databases were imported into the Covidence systematic review management system, where duplicates were then removed. In addition, articles were stored in a referencing manager known as Mendeley. Titles and abstracts of the retrieved studies were independently screened for inclusion by two reviewers (C.J. and S.O.). Thereafter, the full texts of all potentially eligible papers were reviewed independently by the same reviewers before making a final decision on eligibility. Any discrepancies were discussed until a decision was reached. A third reviewer (L.L.) acted as an adjudicator if a decision could not be reached.

### 2.2. Study Inclusion and Exclusion

Studies were included if they met the following criteria: peer-reviewed articles, full-text studies, participants who are undergraduate university students or young adults aged 18 years and older, articles focusing on PA and exercise, articles written in English, articles published from 2011 to 2022, as well as articles published worldwide. Studies were excluded if they were non-peer-reviewed; they were not available in full text; participants were not at a university, were enrolled in postgraduate studies, were older adults (65 years and older), or were children and teenagers/adolescents; the articles focused on physical education; the articles were not published in English; the articles were published before 2011; or they were unpublished.

### 2.3. Data Extraction

Data were extracted by two reviewers (C.J. and S.O.). This was conducted independently using a data extraction form (Supplementary File S2) that was developed prior to the research. The following data were extracted: author(s), date of publication, country, sample characteristics (size, age, and sex), setting, study design, study objectives, duration, strategies such as the data collection tools for PA, best practices, study outcomes, and study conclusions. This information provided a description of the included studies. A third reviewer (S.T.) acted as an adjudicator to resolve any conflicts. The data were synthesised in a narrative approach. Extraction was checked for accuracy and consistency by a third reviewer (S.T.).

### 2.4. Quality Assessment

The included studies were evaluated by two independent reviewers (C.J. and S.O.) using the Critical Appraisal Skills Programme (CASP) and the Reach, Effectiveness, Adoption, Implementation, and Maintenance (RE-AIM) frameworks. Research has demonstrated that the CASP tool for qualitative, quantitative and mixed methods, and cohort studies [26] and the RE-AIM framework [27] for random controlled trials are critical appraisal assessment resources that are relevant and dependable. All studies meeting the inclusion criteria underwent quality assessment using the adapted CASP and RE-AIM appraisal tools. Specifically, the RE-AIM tool focused on the following categories to determine the nature of the study and how the intervention was implemented:

*Reach:* The study described the intended and target population that was reached; the eligibility criteria; inclusion and exclusion of the sample; and the total number of participants who engaged in the study.

*Efficacy:* The effectiveness of the intervention was determined by the effects of the intervention regarding participation in PA.

*Adoption:* The adoption of the study was determined by the location of the intervention, the accessibility of the research setting, and the openness of participants to engage in the intervention.

*Implementation:* An evaluation of the execution of the intervention was conducted to determine if the study obtained the research objectives and to confirm the suitability of the research methods.

*Maintenance:* The maintenance of the intervention was determined by the long-term effects on university students over time, such as >6 months.

The quality of the studies were rated using percentage scores based on the content of the articles. Each article was evaluated and rated according to a three-grade scale: good (67–100%), satisfactory (34–66%), and weak (0–33%). The third reviewer (S.T.) acted as an adjudicator to resolve any conflicts.

The database search yielded a total of 32,052 entries (Figure 1). From these, 11,622 duplicates were removed. Of the remaining 20,430 studies in the title and abstract screening, 20,222 were found to be irrelevant. This therefore left a total of 208 full-text studies that met the inclusion criteria. The full texts were then screened for final inclusion in the review and a total of 197 studies were excluded. The reasons for exclusion included studies not being in a university setting (*n* = 67), no mention of a PA strategy or best practice (*n* = 53), studies that focused on unsuitable methodologies such as systematic and narrative reviews (*n* = 49), studies focusing on an unsuitable target group (*n* = 24), studies that were not in an article format (*n* = 2), studies that were not written in English (*n* = 1), and studies that were not available in full text due to subscription fees (*n* = 1). After excluding the articles, 11 studies were suitable for inclusion in this systematic review.

## 3. Results

### 3.1. Descriptive Characteristics of the Included Studies

The eleven studies included in this review employed the following research designs: two cross-sectional, two quasi-experimental, one non-randomised controlled trial, one experimental, one factorial, one prospective longitudinal, one interventional, one repeated measure, and one where the study design was not specified. Studies were conducted between 2012 and 2021 and included a total of 1271 university students.

A summary of the included studies can be found in Table 1. The detailed critical appraisal of each study may be found in Supplementary Table S1 (quantitative studies) and Supplementary Table S2 (RCTs).

Regarding demographic information from the included studies, two solely focused on female students as a sample group [28,29]. One study did not indicate the gender of the sample [36], whereas eight studies comprised both male and female student samples (although among these samples, females were the predominant participant group). In terms of age, the mean age across the studies ranged from 18 to 25 years of age, and one study did not indicate the mean age [34]. From the included studies, 64% (seven out of eleven) included undergraduate students, whereas four studies did not specify the student population. More specifically, one study focused solely on medical students [36], two articles focused on first-year students [33,34], and one study specified their sample’s race (Asian, black/African American, white, and multiracial) [30]. Studies took place at various universities located across different regions of the world, including Saudi Arabia [28], the United States [29,30,37,38], China [31], Belgium [32], the UK [33], Serbia [34], India [35], and Ireland [36]. 

### 3.2. Physical Activity Levels

In terms of PA levels, 73% of the articles (8 out of 11) reported on an improvement in PA engagement [30,31,32,33,34,35,36,37]. These results indicate that the best practices for enhancing PA levels included the use of mobile phone applications (Google FIT, WeChat, and exergaming), social media platforms (WhatsApp, Facebook text messages, and SMS), web-based platforms (Zoom and Google), and the more conventional “class-like” traditional method. Three studies (27%) indicated that adhering to PA was poor among female students [28], had a limited effect [38], and did not find any increases in perceived PA [29].

### 3.3. Strategies for Enhancing Physical Activity

The monitoring period of the respective interventions lasted approximately between two weeks and two months. Strategies to improve participation in PA involved various approaches, such as the data collection methods, used to generate a plan to enhance engagement among students. Throughout the 11 studies in this review, various strategies were used to measure PA levels. The majority (73%) of studies measured PA levels subjectively by utilising questionnaires [28,29,30,31,32,33,34,36,37,38].

The International PA Questionnaire Short Form (IPAQ-SF) [32,33] and International PA Questionnaire Long Form (IPAQ-LF) [34] were the most frequently used measurement tools. The IPAQ-SF is a self-reported questionnaire that assesses PA across seven days and consists of seven items that focus on weekly time spent in vigorous activity, moderate activity, and walking [39]. These categories are calculated by multiplying the frequency and duration reported in each activity category. The total weekly time spent in PA is determined by summarising the three categories of activity listed above. In addition, this questionnaire examined sitting time [39,40]. The level of PA is quantified using the metabolic equivalent of task (MET) min/week as the measure in the IPAQ-SF classification. The MET min/week is a physiological measure expressing the energy cost of physical activities. This is defined as the ratio of metabolic rate (and therefore the rate of energy consumption) during a specific PA to a reference metabolic rate, usually represented by the resting metabolic rate [16]. The variable MET min/week expresses weekly metabolic engagement in vigorous, moderate, and walking physical activities [16]. The IPAQ-LF [40] is a 27-item questionnaire and classifies PA not only by the intensity but also by the context in which the activity is performed, such as work, transportation, domestic and gardening activities, and leisure time. Studies indicated different timepoints of when the questionnaires were distributed, such as one week prior to the start of the intervention and one week after completion [30], at baseline and once again after one month [34], and pre- and post-programme intervention with four weeks in-between [36]. Additional questionnaires consisted of the Paffenbarger PA Questionnaire [29], Exercise Motivation Inventory-2 [28], Physical Activity Readiness Questionnaire [37], and Physical Activity Questionnaire [38]. From the included studies, three measured PA objectively by making use of heart rate monitors [32], pedometers [35], and accelerometers [37].

Objective strategies used to measure PA utilised anthropometric parameters such as measuring height, weight, BMI [28], heart rate monitors, VO_2_, cadence [32], the Bruce treadmill protocol, and step-counts [35], as well as accelerometers [37]. Height was measured using a fixed stadiometer and weight was measured with the Beurer glass diagnostic scale [28]. Participants utilising heart rate monitors were asked to breathe through masks to record their heart rate. Heart rate was recorded via a telemetry monitor. Participants’ VO_2_ was recorded via indirect calorimetry using a calibrated metabolic cart [32]. In terms of the Bruce treadmill protocol, all participants were familiarised with the treadmill walking and study protocol before administering the baseline measurement. Students had to walk or run on the treadmill at incremental speeds and inclinations [35].

### 3.4. Best Practices for Enhancing Physical Activity

Best practices focused on the type of intervention found to be the most effective in enhancing PA participation levels. Studies included in this review applied various practices to enhance the PA levels of students in the university environment. These best practices have been classified into three overarching categories and are fully described in Table 2, namely social network sites (consisting of social media, web-based technology, mobile phone application, and online text messages), classes, and exercise games (exergames).

In terms of social network sites, the majority of the studies (73%) made use of media technologies such as social media platforms [28,29,30,31,34,35], text messaging [33,35], and mobile phone applications [32,38] as innovative methods to deliver PA practices. This included platforms such as Facebook, Instagram, YouTube, Zoom, Google, WhatsApp, and short message service (SMS). Studies by Todorovic et al. [34] and Al-Eisa et al. [28] were similar in the sense that both focused on participant interaction through a social media platform. Todorovic and colleagues [34]) utilised a Facebook group specifically designed for participants to post motivational messages and questions for their peers. In addition, reports and photographs were posted on the group for fellow students to view and to follow the PA status of their friends [36]. Meanwhile, Al-Eisa et al. [28] made use of Instagram to conduct a similar intervention. Motivational pictures and educational content on the benefits of regular PA were posted. Students were encouraged to motivate each other by posting their own pictures of their participation in PA.

Throughout the studies, there were common categories of PA practices, but the manner in which these interventions were conducted differed. For example, Mo et al. [31] organised participants into peer-support teams involving five to six members, with the aim of creating cohesion among teammates and to promote friendship, accountability, competition, and social engagement. This enabled a platform for peers to encourage each other to achieve their daily duration goal for PA [31]. Another study administered six messages, which were sent on various days (i.e., Monday, Tuesday, Thursday, and Saturday) at various times (such as 9 a.m. and 2 p.m.) [33].

In the study by Cavallo et al. [29], participants were provided with access to a health promotion website, which provided educational information related to PA. This website had a built-in self-monitoring tool that allowed participants to set goals, track their PA levels, and view their progress in relation to their goals. A Facebook group was created where participants were able to exchange social support to encourage participation [29]. Marenus et al. [30]) used the web-based Zoom platform, where participants attended two 30 min exercise lessons per week. A certified student instructor administered the exercise lesson. Participants had the choice of attending the live lessons or viewing the recording of the exercise programme.

Two studies in this review focused on steps per day [35,38]. In the study conducted by Tulasiram and colleagues [35], participants made use of a smartphone Google application to record their daily steps. All participants were provided with weekly feedback on their progression via SMS and WhatsApp. Zhang et al. [38] used the built-in accelerometer feature of smartphones to count and record the participants’ steps.

Regarding classes, one study focused on a lecture-like method. The combined exercise and educational intervention designed to promote well-being among medical students was known as the MED-WELL programme [30]. This intervention was administered through six weekly sessions lasting one hour each. A healthcare professional spoke for 15 min on an exercise topic. The remaining 45 min consisted of PA adapted to different ability levels by a professional instructor [36]. The six-week MED-WELL programme schedule consisted of the following. Week 1: sports yoga focusing on exercise as a medicine; week 2: high-intensity interval training focusing on practical applications of PA; week 3: body pump focusing on the importance of PA; week 4: Pilates focusing on overcoming resistance; week 5: Pilates with a respiratory focus involving motivational aspects; and week 6: sports yoga using a drumbeat focusing on behaviour change.

In terms of exercise games, also known as exergames, Yang and colleagues [37]) administered cardio exercises in the form of an exergame. This study focused on 15 min sessions of PA with a 5 min rest period before commencing with the next session. Similarly, another study used a mobile application-based exergame, where each participant was required to complete an incremental cycling test on a bicycle ergometer [32].

### 3.5. Critical Appraisal of Randomised Controlled Trials

Regarding the critical appraisal of the included studies, three randomised controlled trials [29,33,35] were critically apprised according to the RE-AIM framework. These three studies are described and presented below in accordance with the RE-AIM framework.

*Reach:* All three interventions targeted participation in PA. Participants were recruited through electronic communications including email [29,33], Facebook [29,35], Twitter [29], posters [33], and advertisements [35]. The inclusion criteria were reported and included being currently enrolled at the university under examination, age, gender, reporting <30 min of daily PA and >30 min daily use of Facebook, being a first-year undergraduate student, and owning a mobile phone (specifically an Android smartphone). All three studies referred to their exclusion criteria, which were mainly a result of severe medical conditions such as taking medication (e.g., the presence of anti-anxiety or anti-psychotic drugs); having undergone a recent surgery; cardiovascular, pulmonary, and neuromuscular diseases affecting functional capacity; and disordered eating. The total enrolment across the three intervention studies was 452 students. All three studies reported on their participation or retention rate. In the study conducted by Cavallo et al. [29] all 134 students participated. St Quinton et al. [33] (indicated that 289 participants completed the baseline questionnaire, 179 (61.94%) responded to the follow-up questionnaire, 169 (58.48%) to the second follow-up questionnaire, and 135 participants completed all three questionnaires. However, in the study conducted by Tulasiram et al. [35] from a total of 77 participants recruited, only 26 undergraduate students with a step count of less than 7500 steps/day completed one of the two interventions. None of the interventions reported on methods to retain participants.

*Efficacy:* Two intervention studies indicated that their objectives were met [33,35]. However, Cavallo et al. [29] did not meet their intended objectives. Although their study suggested that online social networks are a feasible platform for intervention delivery, the results did not find an increase in perceived social support or PA. Table 2 describes the effectiveness of the interventions. Interventions were effective because of messages successfully influencing attitude, intention, and behaviour, where changes in behaviour were mediated by changes in attitude and intention, with attitude influencing intention [35]. Furthermore, Tulasiram et al. [35] suggested that employing a smartphone application for prescribing PA is more effective in enhancing participation compared to the conventional approach of exercise prescription and the promotion of PA. All three articles provided recommendations to improve the intervention.

*Adoption:* Interventions were adopted in the United States (at a Southeastern public university) [29], the UK (across 57 universities) [33], and India (within the Department of Exercise and Sports Science of the Manipal Academy of Higher Education) [35]. Interventions were administered and conducted by primary researchers and moderators and were conducted online through social networks such as Facebook [29], via text message [33], and using smartphone applications [35]. All three studies reported having consultations to partner with the university prior to the intervention, and ethical clearance was obtained.

*Implementation:* The implementation of the interventions varied across the three studies. Interventions were implemented through various methods such as online practices and face-to-face methods. Training was required to implement the intervention. Two studies reported prior training of the facilitators before commencing with the intervention [29,35]. The moderators’ role was to encourage the participants and to answer technical or other questions related to PA [29]. Meanwhile, the primary investigators’ responsibility was to collect the snapshots of the participants’ weekly average step count [35]. In addition, the primary investigator familiarised their study participants with the treadmill protocol before administering the baseline measurement. None of the three studies indicated the duration of training that was required for the moderator and the primary investigator to fulfil their roles. Resources that were required to conduct the intervention consisted of utilising the internet/website [29], posters [33], treadmills [35], and a portable metabolimeter [35]. Intervention time ranged from 4 weeks to 12 weeks for the period of implementation. In the intervention conducted by Cavallo et al. [29] (participants had to log into the Internet Support for Health Associations Promoting Exercise (INSHAPE) website on average approximately every two weeks during the intervention. One intervention administered a total of six text messages that were sent on various days (i.e., Monday, Tuesday, Thursday, and Saturday) and at various times (i.e., midday, 9 a.m., 2 p.m.) throughout the two-week intervention period [33]. Another study indicated that their participants were required to participate in physical activities every day for 30 min for four weeks [35]. All study participants across the three studies provided consent to engage in the PA interventions. Only one study reported feedback in the form of a post-study survey. Their results reported that 66% of survey respondents indicated that they would recommend the programme to their friends [29].

*Maintenance:* In the RE-AIM framework, maintenance refers to the long-term effects of the intervention and whether the outcomes were maintained after six months post-intervention [27]. Nevertheless, it is important to consider short-term follow-up methods as this indicates if the intervention met the objectives [41,42]. One study reported utilising a follow-up questionnaire [33] and two follow-up questionnaires were administered approximately four weeks apart. The results showed a significant effect regarding attitude messages; specifically, attitude messages had a significant effect on attitude, intention, and PA. As a result, participants receiving attitudinal messages had a more positive attitude and better intentions, as well as higher levels of PA. Two studies did not conduct a follow-up but rather recommended that for accurate measurement and to influence adherence to long-term PA, a larger sample size should be targeted [35]. One study reported on their attrition rate (the percentage of participants at baseline who participated in the follow-up), indicating that attrition was different between the intervention group (16%) and the control group (4%) [29]. According to the findings reported from the three studies, two studies indicated that using online methods is effective for enhancing engagement in PA [33,35]. However, another study indicated that participants were satisfied with the use of Facebook as a platform for intervention delivery but that it was not effective in perceived PA [31].

Based on the findings presented in this review, all 11 studies emphasise strategies and best practices designed to increase the PA levels of university students. These strategies include the development of tools to measure PA, with the goal of enhancing overall participation. Additionally, the review identifies best practices for improving PA, categorised into four themes. Further discussion of these strategies and best practices aims to provide valuable insights for the enhancement and advancement of PA levels.

## 4. Discussion

The objective of this review was to present a systematic review that focuses on the strategies and best practices used to enhance the PA levels of undergraduate university students. The results derived from this study provide an overview of existing strategies and best practices, detailing their implementation and suggesting ways to improve them in order to overcome the worldwide public health issue of reduced PA levels among young adults. Based on the findings of this review, the following strategies and best practices enhanced PA participation (statistical outcomes of PA interventions may be seen in Table 2). Strategies included implementing validated PA questionnaires, such as the IPAQ-SF, IPAQ-LF, and PAR-Q, and utilising objective measures like heart rate monitors, calorimetry, cadence, the Bruce treadmill protocol, and accelerometers; these studies aimed to enhance the PA levels of university students. Regarding best practices, the utilisation of social network sites, including web-based technology, social media platforms, mobile phone applications, online text messaging, as well as a lecture-like PA class and an exergame, represents the diverse approaches considered effective in promoting PA participation.

### 4.1. Physical Activity Levels

In line with previous studies [24,43], the effectiveness of the interventions for enhancing the PA levels of university students varied across the studies (Table 2). It is evident that not all interventions worked equally well for students. Recognising this is therefore important in order to tailor interventions to specific target groups. For example, the findings of this review indicate that the predominant group of participants in the PA interventions were female students, suggesting that they may encounter distinctive challenges in maintaining adherence to PA programmes. These findings are similar to a previous study where it was indicated that female university students may experience PA participation challenges and barriers such as psychosocial challenges including a lack of self-esteem, motivation, and confidence as well as accessibility and safety concerns [14]. Prior studies have suggested that female university students recommend various forms of PA [14]. These include jogging, cardio/aerobic classes, cycling, swimming, and Zumba/dance classes. Participants further emphasised the importance of enjoyable activities led by an instructor possessing qualities that are both entertaining, motivational, and educational [14]. Taking this into consideration, it is important to encourage gender-specific PA strategies to be implemented. Nevertheless, the variety of strategies and best practices used to enhance PA suggests the importance of offering various methods to cater for diverse PA preferences and lifestyles. These results are important as they offer practical insight into how universities could design strategic interventions that could enhance PA.

### 4.2. Strategies for Enhancing Physical Activity

For the strategies such as the measurement tools for PA used in the interventions, the majority of studies (73%) made use of validated PA questionnaires. The IPAQ-SF and IPAQ-LF were the most commonly used questionnaires. Previous research has indicated that questionnaires have been found to be beneficial in comparison to other methods such as pedometers and accelerometers because of the low influence that it has on the results [23]. This may be due to the reliability and reproducibility of results [44,45]. However, a previous systematic review analysed the IPAQ-SF and reported an overestimation of PA by approximately 84% [46]. Accordingly, despite being reliable, the validity of the IPAQ-SF requires further examination [47]. Despite this result, recent studies exploring the IPAQ-SF and IPAQ-LF have researched the use of this tool across languages, countries and various populations including university students [48,49]. Therefore, the results of this systematic review indicated that the IPAQ-SF and IPAQ-LF are useful strategies for monitoring and tracking undergraduate students’ PA levels and to enhance their participation, owing to the generalisability and worldwide recognition of the IPAQ questionnaires.

In conjunction with the PA questionnaires above, studies from this current review aimed to change PA behaviours by using various questionnaires to measure psychosocial determinants of behaviour that influence participation in PA. Various studies in this review targeted psychosocial questionnaires to improve health behaviours. These consisted of the Exercise Motivation Inventory (EMI-2) [28], the World Health Organisation—5 (WHO-5) Wellbeing Index [36], Physical Activity Readiness [37], Enjoyment of Physical Activity [38], and Self Efficacy for Exercise Scale (SEES) [38]. Previous research has indicated that the transition from high school to the university environment often results in lower levels of PA [50,51,52,53]. This could be attributed to the fact that engagement in PA becomes a voluntary decision as young adults develop autonomy at university [54,55,56,57]. As students experience newfound independence, they might not have fully developed self-efficacy and accountability, putting them at a greater risk of adopting sedentary behaviours [43,53,57,58]. Based on these results, it is evident that PA has been linked to determinants of behaviour. As a result, by considering the psychological and social aspects that influence participation in PA, more comprehensive and effective strategies can be developed.

Although the primary focus of the 11 studies concentrated on enhancing PA levels through diverse strategies, these studies also incorporated psychosocial factors and behavioural theories to guide the PA intervention strategies. Two studies incorporated the Theory of Planned Behaviour [31,33] to alter the PA behaviour patterns of students. Furthermore, studies investigated motivation (6 out of 11 articles), social support (5 out of 11 articles) and mental health (3 out of 11 articles). The PA strategies shared similarities such as enabling students to customise their PA preferences, emphasising personalised goal-setting, delivering tailored motivational and encouraging messages, and, lastly, providing individual-specific feedback on activity levels.

The current results were supported by the previous literature which indicated that by applying self-monitoring interventions, such as tailored feedback, to the field of PA, there is a potential for people to enhance their PA levels [59]. Previous systematic reviews have indicated that self-monitoring, goal-setting, self-recording, and feedback procedures were commonly used interventions to promote PA engagement and to modify behaviour among university students [59,60]. By incorporating these components, students are able to establish and maintain healthy habits and active lifestyles, which may have a positive impact on their overall well-being.

Considering the results obtained from this review, it is worth highlighting that incorporating psychosocial instruments and theories may be beneficial components to understanding PA behaviour. For example, the Self-Determination Theory (SDT) identifies three core psychological needs such as autonomy, competence, and relatedness [61]. In the context of PA, SDT suggests that people are more likely to engage in and sustain engagement levels when it aligns with these needs. Autonomy involves feeling in control of fitness choices, competence relates to skill development, and relatedness focuses on positive social interactions [61,62]. Both intrinsic motivation, driven by internal enjoyment, and extrinsic motivation, such as external rewards, are determinants of behaviour that play a role in PA participation [62]. By recognising the underlying determinants of PA behaviour, primary researchers would be able to investigate strategies to enhance PA that are tailored to undergraduate university students. Moreover, providing students with feedback on their PA levels and granting them the ability to personalise their goals empowers them to take responsibility, assess their involvement, and oversee their health progress.

### 4.3. Best Practices for Enhancing Physical Activity

Regarding the best practices for enhancing PA levels, diverse techniques that were implemented in the university environment were grouped into three categories, namely social network sites (consisting of social media, web-based technology, mobile phone applications, and online text messages), classes, and exercise games (exergames). Modern technologies such as social media, applications, and text messaging have influenced many aspects of our daily lives, ultimately influencing how we communicate and access information. Digitally inclined individuals such as university students have the potential to utilise technology in a manner that transforms social media, applications, and/or text messaging into valuable tool(s) to promote engagement in PA [60]. From the studies included in this review, 91% reported using contemporary and innovative methods—including web-based technology, text messaging, mobile phone applications, exergames, and social media—to address physical inactivity, except for Worobetz et al. [36] who made use of a “class-like” traditional method.

With the social network sites, the results of this systematic review indicated that the majority of studies (73%) made use of media technologies such as social media platforms [28,29,30,31,34,35], text messaging [33,35], and mobile phone applications [32,38]. These social networking platforms consisted of Facebook, Instagram, YouTube, Zoom, Google, WhatsApp, and SMS. These results concur with previous systematic reviews, where it was indicated that technology such as mobile phone applications [63] and social networking platforms [60] are effective practices for improving the health behaviours of students. Research has also revealed that social channels have become prevalent platforms for student communication, information sharing, and learning [64] since information is easily accessible, engaging, and visually appealing [65,66]. Social networking sites are user-friendly among students since there is a sense of familiarity with digital platforms [67]. Students use these sites on a daily basis for social interaction and entertainment. This familiarity may make it easier for students to adopt PA routines that are convenient and easily accessible [67]. Based on these results, social network sites may be considered as a best practice to enhance the PA levels of undergraduate university students.

From the studies included in this review, only one administered a “class-like” traditional method [36]. This study involved a six-week programme of 1 h long sessions, starting with a 15 min lecture and a 45 min PA task. Although this PA practice differs from the social networking platforms mentioned above, certain components of the intervention were similar. This “class-like” method was similar due to the fact that not only did the PA intervention focus on enhancing PA levels (by using yoga and Pilates) but it also addressed mental and social health.

The findings from this study are similar to previous systematic reviews focusing on PA interventions that target university students. A previous systematic review suggested that in-class lecturing with interactive face-to-face learning remains the most common teaching method used for educational purposes [20]. This previous review indicated that interventions were conducted within lecture-room settings and included utilising educational programmes, courses, workshops, and seminars, with the majority of them extending over a single academic semester. These PA interventions predominantly employed teaching methods such as lecturing, practices, group discussions, problem solving, assigned homework with feedback, as well as peer training, where qualified students (peers) offered education and guidance [20]. Similarly, Maselli and colleagues [24] indicated that PA sessions guided by an expert may be important in supporting behavioural change techniques. Based on these results, it is important to note that although digital methods are being incorporated in the university setting to enhance PA, class-like lectures that are educational, instructional, and experiential are fundamental for learning. This would allow the student to practice and learn from observation and experience, guided by an expert in the field of PA and exercise [24].

Previous research has indicated that exergaming may be an effective practice to promote engagement in PA [66]. Exergames combine video gaming with PA, which helps to make exercise more entertaining and accessible. This evolving digital practice provides an alternative to traditional PA, making it more appealing to contemporary university students [68,69]. In the present study, only two articles focused on exergames (32.37). Yang et al. [37] used an Xbox Kinect gaming console consisting of the Xbox 360 video game console and a self-adjustable camera that acted as a sensor to detect whole-body movements. This exergame allowed the participants to control the games using their body movements. However, Roure et al. [32] made use of a different exergame practice called Greedy Rabbit. Their study focused on a design-based bike exergame; participants played either the experimental version or the placebo version of Greedy Rabbit, installed on an iPad and paired with an exercise bike via a Bluetooth protocol. In this study, the experimental group played the exergame Greedy Rabbit, which consisted of a total of 32 stages. Each stage resembled a “pac-man” labyrinth where players controlled a rabbit, avoided computer-controlled hedgehogs, and collected flowers to reach a carrot goal. The rabbit’s speed was linked to their cycling, where the exercise intensity varied among three levels (easy, medium, and hard) based on an individual’s fitness level. Players needed to complete the stages without losing all three lives. The control group also played Greedy Rabbit, but no goal elements such as hedgehogs, flowers, or carrots were involved. The rabbit’s speed was determined by their cycling pace and included having the same exercise intensity options.

The previous literature suggested that although advances in technology have provided many benefits to society, new technology has also led to physical inactivity [70]. However, more recent studies have found that exergaming can increase energy expenditure among university students and ultimately provide an exciting way to increase PA levels [69,71,72]. Based on these results, exergaming may be a useful practice to enhance PA, particularly for university students who have time and resource constraints that make regular exercise challenging [71].

To ensure these strategies and practices contribute to the improvement of PA, it is essential to address the challenges encountered by the interventions discussed in this systematic review. Challenges involved low attrition rates [30], the use of self-reported subjective questionnaires [31], short follow-up periods [32,33,34], no control group [36], and the timeframe of when the PA intervention was conducted [36]. These best practices could be improved by implementing attrition measures to inform adherence to the study protocol [30], include follow-up observations where the positive effects of PA can be maintained over time [31], employ longer follow-up periods and objective assessments of PA [31,34], involve control groups to accurately investigate PA engagement [36], and lastly, to restructure the PA intervention into the university curriculum to increase student participation and to monitor and evaluate the PA levels [36]. In addressing these challenges and implementing the suggestions to improve the interventions, more refined practices could be designed to enhance PA engagement.

### 4.4. Strengths and Limitations

To the best of the author’s knowledge, this is the first systematic review to assess the strategies and best practices that enhance the PA levels of undergraduate university students. The strengths of the present study include the application of the PRISMA checklist [25] in order to guarantee the highest level of accuracy when reporting. The broad inclusiveness of this study, such as using nine electronic databases and including studies from qualitative, quantitative, and mixed-method designs, increases the potential and generalisability of the results. However, some limitations exist which should be acknowledged. First, the key terms “young adults” AND “interventions OR programmes OR programs OR approaches” were included in the search strategy as the original terms “undergraduate students”, “university students” AND “strategies” and “best practices” did not yield enough relevant results after the initial title and abstract screening. The search strategy was therefore adapted and incorporated key terms such as “undergraduate students” OR “university students” OR “young adults” AND “strategies” OR “best practices” OR “interventions” OR “programmes” OR “programs” OR “approaches”. Second, only articles written in English and published from 2011 onwards were included in this review; therefore, key articles related to the objective of this review may have been excluded. Third, the study’s focus on “young adults”, specifically using terms like “undergraduate students” and “university students” may limit the generalisability of findings to other age groups or educational contexts. Lastly, initially the Johanna Biggs Institute (JBI) critical appraisal checklist for systematic reviews and research synthesis was considered in this study’s systematic review protocol. However, this tool was replaced with the CASP and RE-AIM frameworks. The CASP and RE-AIM frameworks were more suitable for the critical appraisal of quantitative and RCT studies, which were included in this review, whereas the JBI focused on analysing systematic reviews. 

### 4.5. Recommendations

All the studies included in this review showcased their strategies and best practices to increase the PA levels of university students. Although the included articles provide valuable information, an opportunity exists to develop PA strategies and best practices in Africa. In the present study, none of the interventions investigated or evaluated how PA can be enhanced in the African region. Therefore, an opportunity exists for researchers to utilise this information and develop strategies and best practices that may be suitable and feasible.

Regarding future research, investigators may consider assessing the effectiveness of innovative PA education and promotional strategies, utilising social network sites to motivate and enhance PA levels. This could involve utilising sites that are frequently explored such as social media platforms, websites, and mobile phone applications. By investigating the effectiveness of these interventions, researchers could design and assess context-specific PA campaigns tailored to the contemporary student.

In terms of monitoring and evaluation, research initiatives may focus on effective monitoring and evaluation procedures to measure the effectiveness of the PA intervention. Physical activity levels are known to fluctuate or decrease at various points in a university student’s journey. It is therefore crucial to monitor when PA levels fluctuate and when students are at risk of leading sedentary lifestyles. By understanding these time periods of PA fluctuation or decrease, researchers could refine their strategies and best practices.

Future research should consider investigating the reasons why undergraduate university students are showing changes in PA behaviour. A comprehensive exploration of these factors may enable student-tailored PA strategies and practices to be developed by universities. Understanding the motivations, challenges, and influences shaping PA behaviour among undergraduate university students will contribute valuable insights for the formulation of targeted interventions aimed at fostering enhanced PA participation.

Furthermore, future research should focus on supporting students’ basic psychological needs, as the fulfilment of these needs correlates with increased PA levels. Additionally, interventions could be developed to guide university staff in becoming more need-supportive towards their students.

## 5. Conclusions

This study supports the previous findings which suggest that undergraduate students need to engage in PA to maintain holistic well-being throughout their journey at university. The current review aimed to explore the strategies and best practices that enhance the PA levels of undergraduate university students. Findings from this review indicated that validated questionnaires emerged as the predominant measurement tool. The emphasis on validated questionnaires suggests a standardised and evidence-based approach, highlighting their role as an effective strategy for enhancing PA. Furthermore, the frequent use of social network sites served as a best practice for implementing and promoting PA interventions. The combination of validated questionnaires and the strategic use of social network sites represents an approach to advancing evidence-based physical activity interventions. Therefore, universities are encouraged to promote health-enhancing physical activities based on the current trends and strategies, such as social media and web-based technology, that are relevant to contemporary university students.

## 6. Patents

### Protocol Registration

Details of the protocol for this systematic review were registered on Open Science Framework (protocol reference: 10.17605/OSF.IO/BV73G).

## Figures and Tables

**Table 1:** Characteristics of the studies included in the systematic review.

Author(s)	Year Published	Country of Study	Sample Characteristics	Study Setting	Study Design
Al-Eisa et al. [28]	2016	Saudi Arabia	58 undergraduate students only females mean age = 20.3 years	University	Quasi-experimental
Cavallo et al. [29]	2012	United States	134 undergraduate students only females aged <25 years	University	Specific design not indicated
Marenus et al. [30]	2021	United States	77 students 61 females, 6 males, and transgender and gender-nonconforming students mean age = 23.4 years	University	Quasi-experimental
Mo et al. [31]	2019	China	52 undergraduate students 27 females and 25 males mean age = 20.7 years	University	Non-randomised Controlled Trial
Roure et al. [32]	2020	Belgium	60 undergraduate students 29 females and 31 males mean age = 20.8 years	University	Experimental
St Quinton et al. [33]	2021	UK	289 first-year undergraduate students 183 females and 106 males mean age = 18.7 years	University	Factorial
Todorovic et al. [34]	2019	Serbia	374 students gender not indicated 144 first-year and 231 fifth-year age not indicated	University	Prospective Longitudinal
Tulasiram et al. [35]	2021	India	29 undergraduate students 22 females and 7 males aged between = 18 and 25 years	University	Interventional
Worobetz et al. [36]	2020	Ireland	72 undergraduate medical students 56 females and 16 males Mean age = 25.0 years	University	Cross-Sectional
Yang et al. [37]	2014	United States	20 students 14 females and 6 males mean age = 20.7 years	University	Cross-Sectional
Zhang et al. [38]	2018	United States	106 students 77 females and 29 males mean age = 20.3 years	University	Repeated Measure

**Table 2:** Strategies and best practices utilised to enhance physical activity levels.

Author(s)	Objectives	Duration	Strategies (Physical Activity Data Collection Tools)	Best Practice	Outcome(s)	Conclusion(s)
Al-Eisa et al. [28]	To investigate the efficacy of using the Instagram application with a home exercise programme as a motivational stimulus in improving physical activity (PA) adherence levels among female college students.	4 weeks	Anthropometric parameters including body weight, height, and body mass index (BMI) were measured. Height was measured using a fixed stadiometer (resolution of 0.5 cm). Exercise Motivation Inventory-2 (EMI-2) questionnaire consists of 51 items that constitute 14 subscales, which provide extensive measures of motivation to engage in PA.	**SOCIAL NETWORK SITE: SOCIAL MEDIA** Use of Instagram with a home exercise programme as a motivational modality.	A total of 47% of students were motivated to gain positive health, with 2% by social recognition and health pressure. Only 4% of the control group was adherent to 4 weeks overall and 17% were adherent in the intervention group. There was a significant difference between the two groups (*p* = 0.04).	The use of Instagram with the home exercise programme as a motivational modality could be attractive and effective to reinforce adherence and maintain an appropriate PA level.
Cavallo et al. [29]	To test the efficacy of a PA intervention that combined education, PA monitoring, and online social networking to increase social support for PA compared to an education-only control.	10–12 weeks	Paffenbarger Physical Activity Questionnaire adapted for online use. Perceived social support for PA was measured using an adapted version of the positive subscales (informational, esteem, and companionship) from Chogahara’s Social Influence on Physical Activity questionnaire modified to explicitly include support experienced through online forms of communication.	**SOCIAL NETWORK SITE: SOCIAL MEDIA** Facebook was utilised to test the efficacy of a PA intervention that combined education, PA monitoring, and online social networking to increase social support for PA.	Facebook participants posted 259 times to the group. No differences were found at baseline between groups with the exception of the Facebook Intensity Scale, t (132) = −2.03, *p* = 0.04, where those in the control group showed higher scores than those in the intervention group.	Participants experienced increases in social support and PA over time but there were no differences in perceived social support or PA between groups over time.
Marenus et al. [30]	To examine the feasibility and effectiveness of aerobic and resistance training (WeActive) and mindful exercise (WeMindful) interventions in improving PA, psychological well-being (PWB), and subjective vitality among college students.	8 weeks	The International Physical Activity Questionnaire- Short Form (IPAQ-SF) (for 15–69 years) was used to measure PA during the last 7 days. It contained 7 items regarding vigorous physical activities (VPAs), moderate physical activities (MPAs), walking, and sitting.	**SOCIAL NETWORK SITE: WEB-BASED** Zoom-based programmes called WeActive and WeMindful. WeActive group attended two 30 min aerobic and resistance training sessions per week, and the WeMindful group attended two 30 min yoga and mindful exercise sessions per week for eight weeks.	A repeated-measures ANCOVA indicated a significant main effect of time for total PA (F = 7:89, *p* = 0:006, η2 = 0:049), vigorous PA (F = 5:36, *p* = 0:024, η2 = 0:022), and walking (F = 7:34, *p* = 0:009, η2 = 0:042) in both intervention groups. The results indicate that both groups experienced an increase in total PA, vigorous PA, and walking after the 8-week interventions.	This study demonstrated that mindful exercise was effective in increasing PA, psychological well-being, and subjective vitality, while aerobic and resistance training might only be effective in increasing PA.
Mo et al. [31]	To test the effectiveness of a PA intervention through a controlled trial by investigating whether it could improve subjectively measured PA and related social cognitive constructs based on the Theory of Planned Behaviour (TPB).	7 weeks	International Physical Activity Questionnaire short form (IPAQ-SF) (for 15–69 years) was used to measure PA during the last 7 days. It contained 7 items regarding vigorous physical activities (VPAs), moderate physical activities (MPAs), walking, and sitting. Daily Physical Activity Duration (DPAD) was recorded on a spreadsheet embedded in WeChat. The participants input their DPAD on the spreadsheet and then posted it to their WeChat groups, so that their PA change over time was monitored by themselves and their teammates. The weekly PA duration for each week was also calculated.	**SOCIAL NETWORK SITE: SOCIAL MEDIA** A social media platform named WeChat was utilised to administer the intervention.	DPAD increased from the baseline to the goal, vigorous physical activity (VPA) time, moderate physical activity (MPA) time, walking time, and sitting time were not significantly different between the groups (*p* = 0.760, 0.823, 0.549, 0.821, 0.050, and 0.553, respectively). Perceived behaviour control, intention, and self-reported VPA and MPA in the intervention group were increased after the intervention, compared with the control group. During the intervention, perceived daily PA duration in the intervention group increased, while it declined in the control group.	The findings indicate that WeChat-based interventions integrating gamification and social incentives could effectively increase subjectively measured PA and related social cognition among Chinese undergraduate students and that it was a promising way to ameliorate the problem of insufficient PA among youths.
Roure et al. [32]	To identify the impact of a design-based bike exergame on players’ PA metrics and Situational Interest.	2 weeks	Heart rate monitor was recorded via a telemetry monitor. VO_2_ was recorded via indirect calorimetry using a calibrated metabolic cart 3. Cadence (rpm) was continuously recorded every second by Vescape^®®^ software.	**SOCIAL NETWORK SITE: MOBILE PHONE APPLICATION** A mobile application-based exergame platform, called Greedy Rabbit.	MANOVA results revealed a significant main effect in players’ PA metrics between both groups, Pillaï Trace = 0.78, F (12.47) = 19.52, *p* < 0.01, η2 = 0.70. The repeated measures ANOVAs determined that mean scores for cadence, % HR max, and % VO_2_ max were higher for the experimental group compared to the control group, except for the % HR max and % VO_2_ max at set 1. No differences were observed between the four sets on %VO_2_ max. Furthermore, all sets did not differ in % HR max except for the first set (73.5 < 76.2, 76.9, and 78.0, *p* < 0.01).	The experimental group reported higher scores for all PA metrics and for two dimensions of situational interest (instant enjoyment and attention demand). Furthermore, the PA metrics increased more during the exergame for the experimental group, reaching the standard guidelines for VPA. This study demonstrated that a design-based bike exergame might be a good option to enhance players’ health-related PA outcomes and situational interest.
St Quinton et al. [33]	To test the effectiveness of attitude and goal priority text messages in promoting students’ participation in PA.	4 weeks	Physical activity was measured at each of the three time-points using three items (e.g., a typical week within the past 4 has consisted of physical activity being performed on at least 5 days).	**SOCIAL NETWORK SITE: ONLINE TEXT MESSAGING** Text messages were distributed to participants using an online text messaging service.	Results showed a significant main effect for attitude messages (F (4, 276) = 5.76, *p* = 0.001, η2 = 0.07). Attitude messages had a significant main effect on attitude (F (1, 279) = 4.12, *p* = 0.04, η2 = 0.01), intention (F (1, 279) = 11.54, *p* = 0.001, η2 = 0.04), and PA (F (1, 279) = 17.06, *p* = 0.001, η2 = 0.05). Marginal means showed participants receiving attitude messages had more positive attitudes (received = 5.64, did not receive = 5.35) and intentions (received = 5.04, did not receive = 4.62), plus greater PA (received = 0.14, did not receive = −0.08) than those that did not receive attitude messages. Goal priority messages had no main effect on the psychological constructs and PA (F (4, 276) = 1.85, *p* = 0.11, η2 = 0.02).	Participants who received attitude messages had significantly more positive attitudes, intentions, and rates of PA. The study provides support for using attitudinal messages delivered via text messaging to influence psychological determinants and PA.
Todorovic et al. [34]	To assess PA level among first- and fifth-year medical students and social media intervention with the aim to improve PA.	Not specified	The International Physical Activity Questionnaire IPAQ-LF consists of a 27-item self-reported measure of PA for use with individual adult patients aged 15 to 69 years old.	**SOCIAL NETWORK SITE: SOCIAL MEDIA** A social media intervention (consisted of motivation for PA through motivational pictures, texts, and discussions).	A total of 85.4% of students were sufficiently active at the baseline, whereas 90.4% were sufficiently active after one month. Multivariate logistic regression analysis showed that students who were part of the Facebook group and students who had sufficient PA at the baseline had a higher likelihood to be sufficiently active after one month.	Social media was valuable in health-promoting interventions and can be used for interventions targeting lifestyle change among young adults.
Tulasiram et al. [35]	To compare the effect of SmPh App (SMART) and traditional American College of Sports Medicine (ASCM) walking prescription on functional capacity on the cardiorespiratory fitness of college-going adults.	4 weeks	The average daily step count was measured using Smartphone-based (SmPh) application “Google FIT”. Maximal aerobic capacity (VO_2_ max) was assessed by administering a modified Bruce treadmill protocol on all participants using an indirect calorimeter.	**SOCIAL NETWORK SITE: MOBILE PHONE APPLICATION** Smartphone walking prescription in improving functional capacity and compliance in college adults (Google—FITT application).	Preference towards SMART intervention was more statistically significant than the ACSM PA intervention (Z = 3.086; *p* = 0.006). The results indicate that smartphone-based PA intervention improves functional capacity (VO_2_ max and VECO_2_) and fatigue levels (treadmill time and anaerobic threshold) more than traditional metabolic calculation-driven PA prescription. After four weeks of walking intervention, there was an average of a 9% increase in VO_2_ in the smartphone-based PA prescription group compared to the ACSM-based PA prescription.	PA prescription using a smartphone application was more effective in improving functional capacity when compared to the traditional way of exercise prescription and PA promotion. Long-term compliance may be better with smartphone-guided exercise prescription.
Worobetz et al. [36]	To determine (1) study feasibility, including recruitment, retention, and assessment of outcome measures, and (2) intervention feasibility, including intervention fidelity, efficacy, acceptability, and potential of medical schools to deliver the intervention.	6 weeks	PA was measured in both questionnaires via a two-part question which asked the participant to record their PA over the last 7 days and over a typical week. The participant was asked how many days they were physically active for at least 30 min at a moderate or vigorous intensity.	**CLASSES: MED-WELL PROGRAMME:** The Medical Wellness (MED-WELL) programme was a six-week programme of 1 h long weekly sessions, each involving a different type of PA (45 min). These sessions were an interactive lecture about how to incorporate exercise theory into daily medical practice (15 min).	Significant improvements were seen in scores after the programme in the WHO-5 Well-Being Index which increased from 63.2 (95% CI: 48–78.4) to 67.5 (95% CI: 55.1–79.9) (*p* < 0.01). Students’ level of PA during a typical week also increased from 3.7 (95% CI: 2.1–5.4) to 4.0 (95% CI, 3.5–4.5) (*p* < 0.05).	It was feasible to deliver an exercise intervention—the “MED-WELL” programme—to educate and promote health and well-being among medical students.
Yang et al. [37]	To determine the amount of time spent in moderate-to-vigorous physical activity (MVPA) during a 30 min bout of exergaming with the Xbox Kinect game console in sedentary college-aged students. A secondary purpose was to examine enjoyment level of participation in the selected exergame.	2 weeks	Treadmill Physical Activity Readiness Questionnaire (PAR-Q): consists of 7 items that screen for evidence of risk factors prior to PA. Accelerometer	**EXERGAME:** An Xbox Kinect gaming console consists of the Xbox 360 video game console and a self-adjustable camera detecting body movements.	Chi-square analysis revealed a significant relationship between level of enjoyment and participation in the Break a Sweat activity, X2 (2, N = 20) = 9.1, *p* < 0.01. A significant relationship also existed between game availability and utilisation as a mode of exercise for future use, X2 (2, N = 20) = 9.1, *p* < 0.01. No relationship existed between game availability and meeting PA guidelines through exclusive game play, X2 (3, N = 20) = 6, *p* = 0.11. The Xbox Kinect game console and game “Your Shape Fitness Evolved 2012—Break a Sweat” activity was successful in maximising time spent in MVPA in sedentary college-aged individuals.	The Xbox Kinect game “Your Shape Fitness Evolved 2012” Break a Sweat activity can be a viable mode of training to achieve the PA Guidelines for Americans in college-aged adults. This Xbox Kinect game may be used effectively as a mode of exercise to meet the PA Guidelines for Americans for this segment of the population.
Zhang et al. [38]	The purpose of this study was to examine the effect of a theory-based smartphone application, efitbuddy, on college students’ PA and motivation.	2 weeks	A self-administered Physical Activity Questionnaire (PAQ) assessed participants’ physical activity. PAQ was a 7-day recall instrument developed to assess general levels of physical activity in healthy youth and adults between the ages of 14 and 20. The Physical Activity Enjoyment Scale (PACES) was designed to measure the positive affect associated with involvement in physical activities in college students, consisting of 16 items. The Self-Efficacy for Exercise Scale (SEES) is a 9-item questionnaire that focuses on the self-efficacy expectations for exercise for adults.	**SOCIAL NETWORK SITE: MOBILE PHONE APPLICATION** An Efitbuddy application on the smartphone.	Males displayed higher PAQ scores compared to females (mean difference = 0.30, *p* = 0.025, d = 0.49) and displayed higher levels of self-efficacy compared to females (mean difference = 1.37, *p* < 0.001, d = 0.80). There were statistically significant moderate and positive correlations among all outcome variables at the pre-test time-point (*p* < 0.001).	The results displayed that efitbuddy has a limited effect on young adults’ PA through a short time period of usage. PA levels and SE scores did not change significantly.

Note: ACSM—American College of Sports Medicine; ANCOVA—Analysis of Covariance; BMI—body mass index; HR—heart rate; MANOVA—Multivariate Analysis of Variance; MPA—moderate physical activity; MVPA—moderate-to-vigorous physical activity; PA—physical activity; SMART—smart phone application; VO_2_—Maximal Oxygen Consumption; VPA—vigorous physical activity.

## Data Availability

All data generated and analysed during this review are included in the published review article.

## Supplementary Materials

The following supporting information can be downloaded at https://www.mdpi.com/article/10.3390/ijerph21020173/s1, Supplementary File S1: Database search strategies; Supplementary File S2: Data Extraction Form; Table S1. Critical appraisal of quantitative studies reviewed (adapted from CASP); Table S2. Critical appraisal of randomised controlled studies (adapted from the RE-AIM framework).
