# Supplementary material for: Strategies and Best Practices That Enhance the Physical Activity Levels of Undergraduate University Students: A Systematic Review
Source: Int J Environ Res Public Health. 2024 Feb 1;21(2):173. doi: 10.3390/ijerph21020173 (PMC10888190; doi:10.3390/ijerph21020173)
Supplement: Supplementary file 1 [file ijerph-21-00173-s001.zip › Supplementary File S1_ Database Search Strategies.pdf]

Supplementary File S1: Database search strategies

Database	Search Terms
PubMed	((((((((((physical activity) AND (exercise)) OR (strategies)) OR (best practices)) OR (interventions)) OR (programmes)) OR (programs)) OR (approaches)) AND (young adults)) OR (university students)) OR (college students)) OR (undergraduate students).
Science Direct	Title and Abstract<br>((physical activity OR exercise) AND (strategies OR best practices OR interventions OR programmes OR programs OR approaches) AND (young adults OR university students OR college students OR undergraduate students)).
Academic Search Complete	Title and Abstract<br>((physical activity OR exercise) AND (strategies OR best practices OR interventions OR programmes OR programs OR approaches) AND (young adults OR university students OR college students OR undergraduate students)).
ERIC	Title and Abstract<br>((physical activity OR exercise) AND (strategies OR best practices OR interventions OR programmes OR programs OR approaches) AND (young adults OR university students OR college students OR undergraduate students)).
Web of Science	Title and Abstract<br>((physical activity OR exercise) AND (strategies OR best practices OR interventions OR programmes OR programs OR approaches) AND (young adults OR university students OR college students OR undergraduate students)).
CINAHL	Title and Abstract<br>((physical activity OR exercise) AND (strategies OR best practices OR interventions OR programmes OR programs OR approaches) AND (young adults OR university students OR college students OR undergraduate students)).
Sage	Title and Abstract<br>((physical activity OR exercise) AND (strategies OR best practices OR interventions OR programmes OR programs OR approaches) AND (young adults OR university students OR college students OR undergraduate students)).
SportDiscus	Title and Abstract<br>((physical activity OR exercise) AND (strategies OR best practices OR interventions OR programmes OR programs OR approaches) AND (young adults OR university students OR college students OR undergraduate students)).
OpenGrey	((physical activity OR exercise) AND (strategies OR best practices OR interventions OR programmes OR programs OR approaches) AND (young adults OR university students OR college students OR undergraduate students)).

ERIC = Education Resources Information Center
CINAHL = Cumulated Index to Nursing and Allied Health Literature
