# Supplementary material for: Strategies and Best Practices That Enhance the Physical Activity Levels of Undergraduate University Students: A Systematic Review
Source: Int J Environ Res Public Health. 2024 Feb 1;21(2):173. doi: 10.3390/ijerph21020173 (PMC10888190; doi:10.3390/ijerph21020173)
Supplement: Supplementary file 1 [file ijerph-21-00173-s001.zip › Supplementary File S2_Data Extraction Form.pdf]

Supplementary File S2: Data extraction form

Author(s)	Year Published	Country of study	Sample Characteristics	Study Setting	Study Design	Study Objectives	Duration	Strategy (Physical Activity Data Collection Tools)	Best Practices	Outcome(s)	Conclusion(s)
