# Supplementary material for: Strategies and Best Practices That Enhance the Physical Activity Levels of Undergraduate University Students: A Systematic Review
Source: Int J Environ Res Public Health. 2024 Feb 1;21(2):173. doi: 10.3390/ijerph21020173 (PMC10888190; doi:10.3390/ijerph21020173)
Supplement: Supplementary file 1 [file ijerph-21-00173-s001.zip › Supplementary Table S1_ Critical appraisal of quantitative studies - Copy.pdf]

Table S1: Critical Appraisal of the quantitative studies reviewed (adapted from CASP) (N = 8)

Author(s)	1. Was the study conducted at a university/college?	2. Is the sampling process clearly stated? (non-probability, probability)?	3. Was the measurement tool valid and reliable?	4. Was the data a primary source?	5. Were the dropout responses reported?	6. Was ethical approval obtained?	7. Were the quantitative methods appropriate?	8. Was the research design correct to address the research question?	Final Score (□/8)	Total (%)
**Mo 2019**	Yes	Yes	Yes	Yes	Yes	Yes	Yes	Yes	7	**100**
**Al-Eisa 2016**	Yes	Yes	Yes	Yes	Yes	Yes	Yes	Yes	8	**100**
**Roure 2020**	Yes	Yes	Yes	Yes	No	Yes	Yes	Yes	6	**87.5**
**Marenus 2021**	Yes	No	Yes	Yes	No	Yes	Yes	Yes	8	**75**
**Worobetz 2020**	Yes	No	Yes	Yes	No	Yes	Yes	Yes	6	**75**
**Todorovic 2019**	Yes	No	No	Yes	Yes	Yes	Yes	Yes	6	**75**
**Zhang 2018**	Yes	Yes	Yes	Yes	No	Yes	Yes	No	6	**75**
**Yang 2014**	Yes	No	Yes	Yes	No	Yes	Yes	Yes	6	**75**
