# Supplementary material for: Strategies and Best Practices That Enhance the Physical Activity Levels of Undergraduate University Students: A Systematic Review
Source: Int J Environ Res Public Health. 2024 Feb 1;21(2):173. doi: 10.3390/ijerph21020173 (PMC10888190; doi:10.3390/ijerph21020173)
Supplement: Supplementary file 1 [file ijerph-21-00173-s001.zip › Supplementary Table S2_ Critical appraisal of randomised controlled studies.pdf]

Table S2: Critical appraisal of randomised controlled studies (adapted from the RE-AIM framework) (N = 3)

	**Cavallo 2012**	**St Quinton 2021**	**Tulasiram 2021**
REACH			
1. Does the article indicate who the intervention is intended for (inclusion criteria)?	Yes	Yes	Yes
2. Does the article report on exclusion criteria?	Yes	Yes	Yes
3. Does the article report on the representativeness of the target population? (gender)?	Yes	Yes	Yes
4. Does the article report on the participation rate?	Yes	Yes	Yes
5. Are there reports on indirect beneficiaries?	No	No	No
EFFICACY			
1. Did the intervention achieve the intended objectives?	No	Yes	Yes
2. Does the article report on the limitations of the intervention?	Yes	Yes	Yes
3. Are there reports of attrition (number of people who completed the programme)?	Yes	Yes	Yes
4. Does the article include recommendations to improve the intervention?	Yes	Yes	Yes
5. Does the article include recommendations for practice?	Yes	Yes	Yes
ADOPTION			
1. Is the setting described in terms of country and place of intervention (e.g., university/college setting)?	Yes	Yes	Yes
2. Is the context described (low socioeconomic, rural, etc.)?	No	No	No
3. Is reference made to how accessible the place of intervention was to the participants (e.g., university/college setting.)?	Yes	Yes	Yes
4. Are there reports on the adoption of the intervention by the participants (e.g., were they open to the intervention, resistant, etc.)?	Yes	Yes	Yes
5. Are there reports on consultation or partnering with the university/college prior to the intervention?	Yes	Yes	Yes
IMPLEMENTATION			
1. Are there reports of resources required to conduct the intervention?	Yes	Yes	Yes
2. Are there reports of who did the intervention (e.g., social worker, teacher, etc.)?	Yes	No	Yes
3. Are the duration and frequency of the intervention described?	Yes	Yes	Yes
4. Is training or experience required to implement the intervention?	Yes	No	Yes
5. Did participants evaluate the intervention?	Yes	No	No
MAINTENANCE			
1. Does the article report on long-term effects of the intervention (e.g., after 6, 12, etc. months)?	No	No	No

2. Does the article report on indicators used for intervention follow-up?	Yes	Yes	Yes
3. Are there reports on the attrition rates (number of those who completed the intervention vs. the number who participated in the follow-up)?	Yes	Yes	No
4. Are there reports of relapse?	No	No	No
5. Is the method of follow-up indicated (e.g., telephone calls, interviews, questionnaires, etc.)?	Yes	Yes	No
**Final Score (□/25)**	**20**	**18**	**18**
**Percentage (%)**	**80**	**72**	**72**
